# SpAHA1 and SpSOS1 Coordinate in Transgenic Yeast to Improve Salt Tolerance

**DOI:** 10.1371/journal.pone.0137447

**Published:** 2015-09-04

**Authors:** Yang Zhou, Xiaochang Yin, Ruijun Duan, Gangping Hao, Jianchun Guo, Xingyu Jiang

**Affiliations:** 1 Institute of Tropical Bioscience and Biotechnology, Chinese Academy of Tropical Agricultural Sciences, Haikou, China; 2 College of Plant Science & Technology, Huazhong Agricultural University, Wuhan, China; 3 Hainan Key Laboratory for Sustainable Utilization of Tropical Bioresources/College of Agriculture, Hainan University, Haikou, China; 4 Department of Biochemistry, Taishan Medical University, Tai’an, China; National Taiwan University, TAIWAN

## Abstract

In plant cells, the plasma membrane Na^+^/H^+^ antiporter SOS1 (salt overly sensitive 1) mediates Na^+^ extrusion using the proton gradient generated by plasma membrane H^+^-ATPases, and these two proteins are key plant halotolerance factors. In the present study, two genes from *Sesuvium portulacastrum*, encoding plasma membrane Na^+^/H^+^ antiporter (SpSOS1) and H^+^-ATPase (SpAHA1), were cloned. Localization of each protein was studied in tobacco cells, and their functions were analyzed in yeast cells. Both SpSOS1 and SpAHA1 are plasma membrane-bound proteins. Real-time polymerase chain reaction (PCR) analyses showed that *SpSOS1* and *SpAHA1* were induced by salinity, and their expression patterns in roots under salinity were similar. Compared with untransformed yeast cells, SpSOS1 increased the salt tolerance of transgenic yeast by decreasing the Na^+^ content. The Na^+^/H^+^ exchange activity at plasma membrane vesicles was higher in *SpSOS1*-transgenic yeast than in the untransformed strain. No change was observed in the salt tolerance of yeast cells expressing *SpAHA1* alone; however, in yeast transformed with both *SpSOS1* and *SpAHA1*, SpAHA1 generated an increased proton gradient that stimulated the Na^+^/H^+^ exchange activity of SpSOS1. In this scenario, more Na^+^ ions were transported out of cells, and the yeast cells co-expressing *SpSOS1* and *SpAHA1* grew better than the cells transformed with only *SpSOS1* or *SpAHA1*. These findings demonstrate that the plasma membrane Na^+^/H^+^ antiporter SpSOS1 and H^+^-ATPase SpAHA1 can function in coordination. These results provide a reference for developing more salt-tolerant crops via co-transformation with the plasma membrane Na^+^/H^+^ antiporter and H^+^-ATPase.

## Introduction

Sodium is a trace element in plants. However, excess sodium in the cytoplasm is toxic to some important physiological and biochemical processes, and efflux of Na^+^ out of the cytoplasm is an important mechanism of salt tolerance. Excess Na^+^ in the cytoplasm can be transported into the external medium across the plasma membrane or into the vacuole across the tonoplast, such that the Na^+^ in the cytoplasm is maintained at a non-toxic concentration. The tonoplast Na^+^/H^+^ antiporter NHX compartmentalizes Na^+^ into vacuoles using the proton gradient generated by vacuolar H^+^-ATPase and H^+^-pyrophosphatase (H^+^-PPase), whereas the plasma membrane Na^+^/H^+^ antiporter SOS1, which is driven by the proton gradient generated by plasma membrane H^+^-ATPases, transports Na^+^ out of cells [1].

NHX proteins from more than 60 plant species have been reported to date [1]. *Arabidopsis* AtNHX1 was the first NHX protein to be described, and its overexpression can significantly improve the salt tolerance of transgenic yeast or plants [2, 3]. Many lines of evidence have indicated that tonoplast-bound NHXs function as Na^+^/H^+^ antiporters and mediate plant salt tolerance by sequestering Na^+^ into vacuoles [4]. As Na^+^ influx into vacuoles via NHXs is energized by the H^+^ gradient created by vacuolar H^+^-ATPase and H^+^-PPase, these two H^+^-pumps should play important roles in plant salt tolerance. Indeed, the salt tolerance of transgenic *Arabidopsis* harboring *AVP1* (vacuolar H^+^-PPase 1) was stronger than that of wild type [5]. Moreover, the co-expression of NHX and AVP conferred stronger salt tolerance to transgenic plants than NHX or AVP alone, a finding that further demonstrates the relationship between NHX and the H^+^-pump, with plant NHX proteins functioning as Na^+^/H^+^ antiporters using the force from the proton gradient produced by the vacuolar H^+^-PPase [6–8]. SOS1 is the only Na^+^ efflux protein at the plant plasma membrane reported to date and functions as a Na^+^/H^+^ antiport system that is driven by the plasma membrane H^+^-pump H^+^-ATPase [9–11], and the salt tolerance of *Arabidopsis* transformed with the *AtSOS1* gene is significantly higher than that of wild type [12]. In addition, plasma membrane H^+^-ATPase activity is involved in plant adaptation to salinity [13]. These findings suggest that the salt tolerance efficiency of the simultaneous expression of SOS1 and plasma membrane H^+^-ATPase genes should be further enhanced, just as the co-expression of the vacuolar H^+^-PPase and Na^+^/H^+^ antiporter results in even higher salt tolerance. However, this hypothesis has yet to be tested. Therefore, the aim of the present investigation was to determine whether the combination of the plasma membrane Na^+^/H^+^ antiporter with H^+^-ATPase effectively increases salt tolerance in transgenic organisms. Herein, a plasma membrane Na^+^/H^+^ antiporter gene, *SpSOS1*, and a plasma membrane H^+^-ATPase gene, *SpAHA1*, were cloned from *Sesuvium portulacastrum*, a halophyte with optimal growth at 200–300 mM NaCl [14, 15]. Functional analyses indicated that SpSOS1 could transport Na^+^ out of cells using the force of the proton gradient generated by SpAHA1. In particular, the co-expression of *SpSOS1* and *SpAHA1* conferred stronger salt tolerance to transgenic yeast cells than the expression of either gene alone. These results may be helpful in developing more salt-tolerant crops through the co-expression of SOS1 and H^+^-ATPase.

## Materials and Methods

### Plant material and cultivation

*Sesuvium portulacastrum* (L.) L. plants, dicotyledonous halophytes belonging to the Aizoaceae family, were collected from the seashore of Haikou, China, and propagated through cuttings (S1 Text). The collection site in the seashore is a wasteland, so no one is responsible for this field. Furthermore, *Sesuvium portulacastrum* grows worldwide and is not specifically exploited or an endangered or protected species. Therefore, no specific permission was required for the collection of the plants. Three stem internodes were cut from the mother plants, incubated in Hoagland solution and allowed to grow at room temperature. After 3 weeks, uniform seedlings were treated with 0, 400, 600 or 800 mM NaCl; three replications for each treatment were performed. The roots and leaves were harvested at various times (0, 3, 6, 9, 12, 24 and 48 h) after NaCl treatment and immediately stored in liquid nitrogen.

### Cloning of *SpSOS1* and *SpAHA1*

Total RNA isolation and cDNA synthesis were performed using a reverse transcription polymerase chain reaction (RT-PCR) kit (Invitrogen, Carlsbad, CA, USA) according to the manufacturer’s protocol. Degenerate primers were designed for *SpSOS1* according to the conserved sequences of plant *SOS1* genes: 5’-ATGAATGATGGGAC(G/T)GC(A/T)AT-3’, 5’-TATAG(A/T/C/G)CCCAA(A/T)GT(A/G)CTTCCATG-3’. To clone the *SpSOS1* gene, the cDNA was used as the template in PCR with the degenerate primers. The PCR product, 1,850 bp in length, is homologous with other plant *SOS1* genes. To obtain the full-length *SpSOS1* sequence, 3’-RACE (rapid amplification of cDNA ends) and 5’-RACE cDNA syntheses were performed with kits according to the manufacturer’s instructions (Invitrogen, Carlsbad, CA, USA). The clones obtained were sequenced, and the overlapping region with the first clone was confirmed. After deducing the open reading frame, gene primers were designed according to the open reading frame (ORF) and including E*co*R I and S*al* I restriction sites (underlined): Forward: 5’-gGAATTCATGGCGGCGTTGACTGAT-3’; Reverse: 5’-ataGTCGACTCATGGTGCAGGGCGG-3’. A fragment containing the ORF was synthesized via PCR from cDNA and sequenced for further confirmation. The *SpSOS1* ORF was cloned into the yeast expression vector p416 GPD to produce the plasmid p416-SpSOS1. Regarding the cloning of *SpAHA1*, an *SpAHA1* EST fragment (GenBank Accession No. DQ925817) was first re-obtained via PCR from cDNA and sequenced for further confirmation. Elongation of the 5’- and 3’- ends of *SpAHA1* was performed using 5’-RACE and 3’-RACE techniques with two pairs of nested primers and cDNA as the template. One pair of primers deduced from the polylinker sequence was from the RACE kit (Invitrogen, Carlsbad, CA, USA), and gene-specific primers were designed according to EST sequences. The 3’-RACE nested PCR primers were 5’-ATTGTCGGTATGACTGGAGATGGTGTG-3’ and 5’-CGTCTCTATTACCATTCGTA TTGTGCTC-3’, and the 5’-RACE nested PCR primers were 5’- TTGGAAAATAGCCCTACTGGTAAGCAC-3’ and 5’- TGAGCATAAATCCGAGCACAATAC-3’. The fragments were cloned into pMD-19T and sequenced. Full-length *SpAHA1* was deduced by overlap of the above two fragments and was obtained via PCR with cDNA as the template and two gene-specific primers (5’-CGGGATCCATGGCCAAGGCCATCAATC-3’; 5’-CGGAATTCCCTTAGACGGTGTAGTTCTGTTGG-3’) and cloned into the yeast expression vector p414 to produce the plasmid p414-SpAHA1. The complete cDNA sequences of *SpSOS1* (accession No. JX674067) and *SpAHA1* (accession No. JX628604) were deposited in GenBank.

### Quantitative real-time PCR

Real-time PCR was performed using a Fast Real-Time PCR System (ABI 7900HT, TaKaRa, Kyoto, Japan). The primers 5’-TTGGCATCGTTGAGGGTCT-3’ and 5’-CAGTGGGAACACGGAAAGC-3’ were used to amplify the housekeeping gene *GAPDH*. Gene-specific primers were designed for a 116-bp fragment of *SpSOS1*; the primers were 5’-GAGCATCTTTGGCAGCACG-3’ in the sense direction and 5’-CTGAGTTTCGGGGATTTTAGG-3’ in the anti-sense direction. A forward primer (5’-GATGGGCTGGTGTCGTTTG-3’) and reverse primer (5’-CCGTAATCCTTCTTGGTGGTG-3’) were designed for a 148-bp fragment of *SpAHA1*. The amplification conditions were as follows: 95°C for 30 s, followed by 40 cycles at 95°C for 5 s and 60°C for 30 s. A dissociation curve from 60°C to 95°C was generated to verify primer specificity, and data analysis was performed using the SDS plate utility software, version 2.4. The mRNA abundances of the *SpSOS1* and *SpAHA1* genes are expressed based on the values measured at 0 h of salt treatment.

### Subcellular localization of SpSOS1 and SpAHA1 proteins

The full-length genes *SpSOS1*, *SpAHA1* or *Arabidopsis SOS1* (*AtSOS1*) without the stop codon at the 3’ end were obtained via PCR and fused in-frame to GFP in the modified binary vector pCAMBIA1300. Primers were designed for *SpSOS1*, *SpAHA1* and *AtSOS1*, as follows: SpSOS1–1300F: 5’-ataGTCGACATGGCGGCGTTGACTGATT-3’, SpSOS1–1300R: 5’-cggGGTACCTGGTGCAGGGCGGGGGAAAG-3’; SpAHA1–1300F: 5’-gACTAGTATGGCCAAGGCCATCAATC-3’, SpAHA1–1300R: 5’-cgGGATCCGACGGTGTAGTTCTGTTGG-3’; AtSOS1–1300F: 5’- gcTCTAGAATGACGACTGTAATCGACGCG-3’, AtSOS1–1300R: 5’- cggGGTACCTAGATCGTTCCTGAAAACG-3’. The resulting constructs were transferred into *Agrobacterium tumefaciens* strain LBA4404. For transient expression in plants, different combinations of strains containing these constructs were co-infiltrated into *Nicotiana tabacum* leaves [16]. After 5 days, protoplasts were isolated from the infiltrated leaves with a buffer containing 0.4 M mannitol, 20 mM KCl, 20 mM MES (pH 5.7), 10 mM CaCl_2_, 3.5 mM β-mercaptoethanol, 0.1% bovine serum albumin, 1.5% cellulase R10, and 0.4% macerozyme R10 [17]. GFP fluorescence in the leaf epidermal cells and mesophyll protoplasts was detected using a confocal laser scanning microscope (FV3000; Olympus Corporation, Tokyo, Japan).

### Yeast strains and incubation

*Saccharomyces cerevisiae* strain AXT3K (△ena1::HIS3::△ena4, △nha1::LEU2, and △nhx1::KanMX4), which is deficient in the main endogenous Na^+^ transporters, is a derivative of W303 (MAT ura3–1 leu2–3,112 his3–11,15 trp1–1 ade2–1 can1–100) [10]. To assess the functionality of the SpSOS1 and SpAHA1 proteins, p416-SpSOS1, p414-SpAHA1 and p416-SpSOS1 and p414-SpAHA1 were transformed into AXT3K. The wild-type yeast strain W303 was used as a positive control [18]. W303, AXT3K, and AXT3K transformed with p416-SpSOS1, AXT3K transformed with p414-SpAHA1, and AXT3K co-transformed with p416-SpSOS1 and p414-SpAHA1 were grown in AP medium (10 mM arginine, 8 mM phosphoric acid, 2% glucose, 2 mM MgSO_4_, 1 mM KCl, 0.2 mM CaCl_2_, and trace minerals and vitamins) [19]. When these precultures grew to saturation, they were diluted 10-fold, 100-fold, 1,000-fold, and 10,000-fold, and then 10 μl of each serial dilution was spotted onto AP plates with the indicated concentration of NaCl. After incubation for 3–5 days at 30°C, growth was imaged and analyzed.

### Measurement of Na^+^ content in yeast cells

Yeast cells were grown in AP medium in the presence or absence of 30 mM NaCl at 30°C. The Na^+^ content in the cells was measured using an atomic absorption spectrophotometer (AA-670, Shimadzu Corporation, Kyoto, Japan), as previously described [19].

### Plasma membrane isolation and measurements of Na^+^/H^+^ exchange and H^+^-ATPase activity

Yeast cells were first cultivated to an OD660 of 1.5 in AP medium. A total of 50 mM NaCl (final concentration) was added to further ensure the activation of SpAHA1 and SpSOS1. At one hour after NaCl treatment, the yeast cells were harvested via centrifugation, and the plasma membrane was isolated using an aqueous two-phase system [20, 21]. The plasma membrane protein content was measured using the Bradford method with BSA as a standard [22]. The activities of H^+^-ATPase and Na^+^/H^+^ exchange at the plasma membrane were determined as described previously using a fluorescence spectrophotometer (Hitachi F-2500, Hitachi, Ltd., Tokyo, Japan) [23]. The reaction mixture consisted of 5 μM quinacrine, 50 mM BTP-HCl (pH 7.5), 25 mM BTP-HEPES (pH 7.5), 50 mM KNO_3_, 250 mM mannitol, 4 mM MgSO_4_ and 50 μg of plasma membrane protein. The assay was initiated by the addition of 3 mM ATP (pH 7.5); when the ΔpH reached a steady state, 50 mM Na_2_SO_4_ was added to the reaction mixture. Finally, the reaction was stopped with 25 mM (NH_4_) _2_SO_4_. H^+^-ATPase activity was assayed by measuring the ΔpH across the membrane, which is expressed as the change in fluorescence quenching during the first 30 s after the addition of ATP (ΔF/min/mg protein). Na^+^/H^+^ exchange activity was calculated as the change in the initial rate of fluorescence recovery during the first 30 s after the addition of Na_2_SO_4_, and this value is expressed as a percentage of the maximal fluorescence (ΔF (%)/min/mg protein). The reaction was controlled at excitation and emission wavelengths of 430 nm and 500 nm, respectively.

### Statistical analysis

A two-tailed Student’s t-test was used to analyze the data, which are presented as the means ± SE.

## Results

### Cloning and molecular description of *SpSOS1* and *SpAHA1*

**Fig 1:**
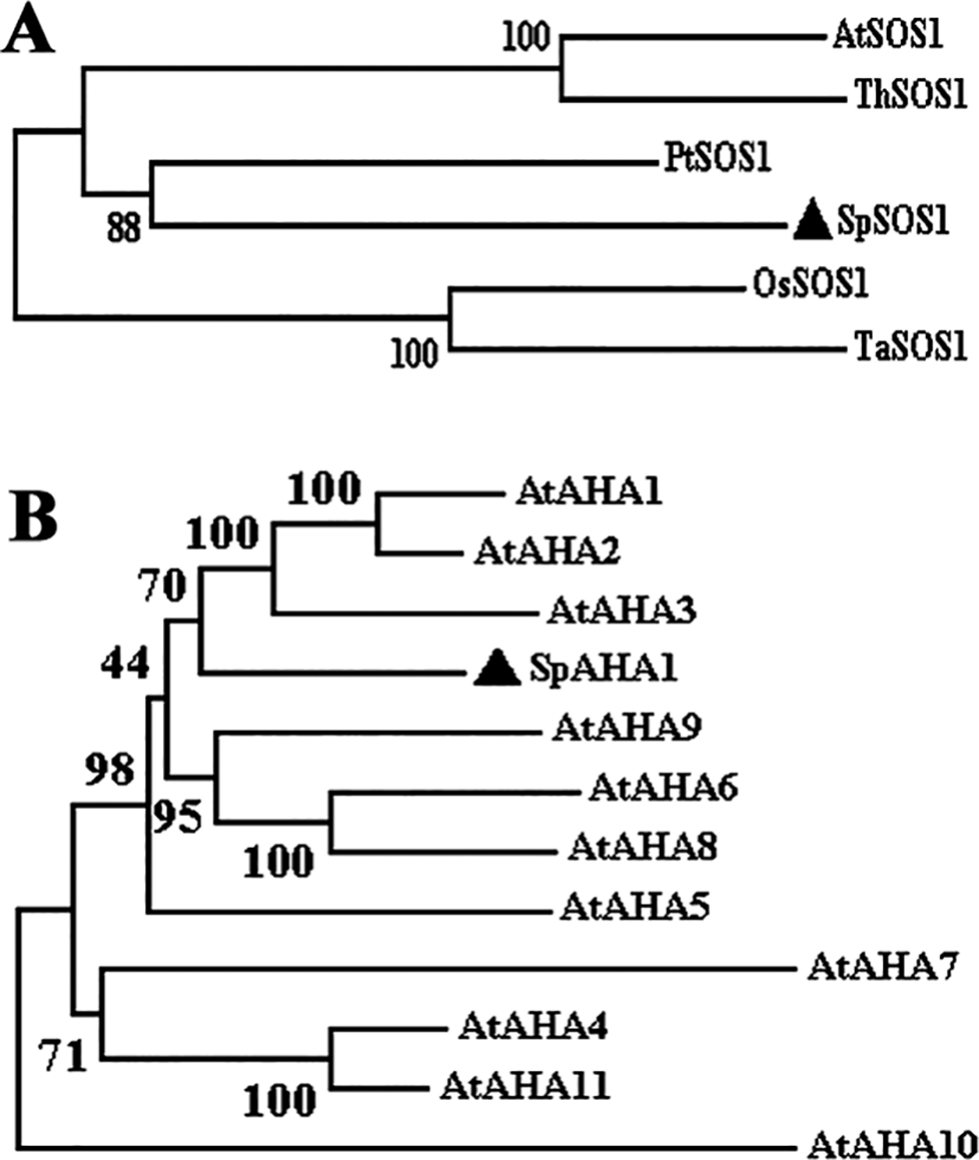
Structural analysis of SpSOS1 and SpAHA1 proteins. (A) Phylogenetic tree of plasma membrane Na^+^/H^+^ antiporters from plants. SpSOS1 (JX674067), AtSOS1 (NM_126259), OsSOS1 (AY785147), PtSOS1 (XM_002315801), TaSOS1 (FN356232) and ThSOS1 (EF207775) indicate Na^+^/H^+^ antiporters from *Sesuvium portulacastrum*, *Arabidopsis thaliana*, *Oryza sativa*, *Populus trichocarpa*, *Triticum aestivum* and *Thellungiella halophila*, respectively. (B) Phylogenetic tree of plasma membrane H^+^-ATPases from plants. SpAHA1 (JX628604) indicates *Sesuvium portulacastrum* H^+^-ATPase. AtAHA1 (NM_127453), AtAHA2 (NM_001203941), AtAHA3 (NM_001203630), AtAHA4 (NM_114664), AtAHA5 (NM_128013), AtAHA6 (NM_126721), AtAHA7 (NM_115897), AtAHA8 (NM_114131), AtAHA9 (NM_001198521), AtAHA10 (NM_101587), and AtAHA11 (NM_125662) indicate *Arabidopsis thaliana* H^+^-ATPases. The phylogenetic trees were constructed using the MEGA5.0 software based on multiple alignments of full-length protein sequences. Evolutionary distances were computed using the neighbor joining method. Bootstrap values are indicated at the nodes of the tree and are expressed as percentages.

To clone the genes encoding the plasma membrane Na^+^/H^+^ antiporter and H^+^-ATPase from *S*. *portulacastrum*, we utilized homologs of other plant plasma membrane SOS1s or H^+^-ATPases and designed degenerate primers. We obtained a fragment of cDNA encoding a putative homolog of the Na^+^/H^+^ antiporter *SOS1* gene, and the complete cDNA was then obtained using the rapid amplification of cDNA end (RACE) method and sequenced (GenBank accession number: JX674067). The homology-based computer search identified a 396-bp EST in *S*. *portulacastrum* (GenBank accession number: DQ925817) encoding a putative plant plasma membrane H^+^-ATPase. The EST was isolated and confirmed via sequencing, and the cDNA was cloned using RACE. The *SpSOS1* gene encodes a protein of 1,155 amino acids. The SpSOS1 protein shares 61.53%, 60.58%, 67.67%, 58.94% and 60.39% amino acid identity with similar proteins from *Arabidopsis thaliana* (AtSOS1), rice (OsSOS1), poplar (PtSOS1), wheat (TaSOS1) and *Thellungiella halophila* (ThSOS1), respectively [11, 19, 23–25] (Fig 1A). TMHMM software analysis revealed that SpSOS1 contains 12 putative transmembrane domains at the N-terminus and a long hydrophilic C-terminus (http://www.cbs.dtu.dk/services/TMHMM/), and a conserved phosphorylation site (1143DSPS1146) is also present at the C-terminus. These findings suggest that SpSOS1 may function as a plasma membrane Na^+^/H^+^ antiporter that is involved in salt resistance in *S*. *portulacastrum*. The 3,394-bp *SpAHA1* cDNA fragment for a putative H^+^-ATPase from *S*. *portulacastrum* was cloned using the RACE method and then sequenced (GenBank accession number: JX628604). This fragment contains a 2,862-bp ORF encoding a protein of 953 amino acids. An alignment of the amino acid sequences of *Arabidopsis* H^+^-ATPases showed that SpAHA1 shares significant similarity with *Arabidopsis* AtAHAs and is closest to AtAHA1 (85.83%) (Fig 1B), indicating that SpAHA1 may function as an H^+^-ATPase at the plasma membrane and assist in energy generation in *S*. *portulacastrum* by catalyzing ATP.

### Subcellular localization of SpSOS1 and SpAHA1 proteins

**Fig 2:**
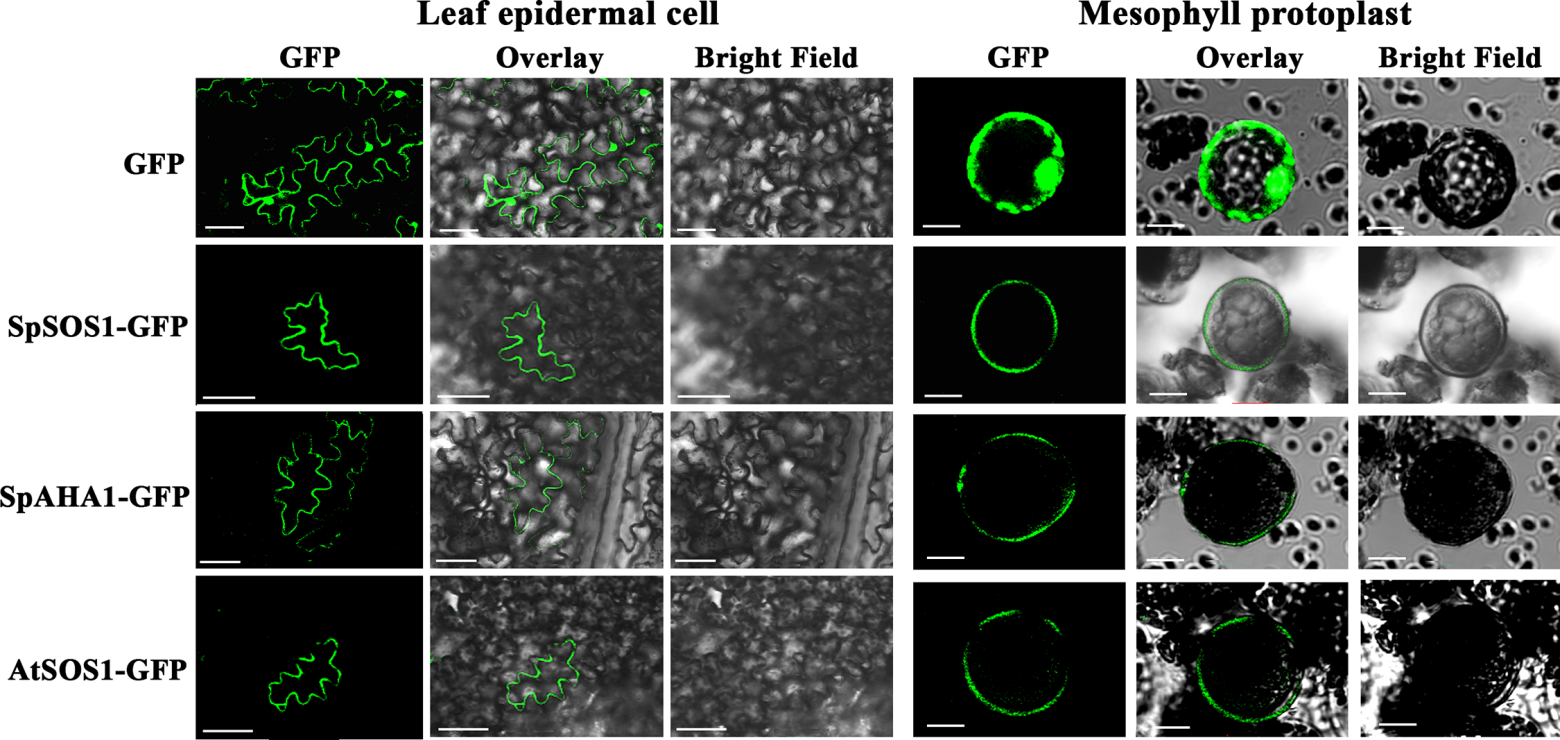
SpSOS1 and SpAHA1 proteins are localized at the plasma membrane. SpSOS1-GFP, SpAHA1-GFP and AtSOS1-GFP fusion proteins were produced as described in the Methods and Materials section. Tobacco leaves were transformed with the vector pCAMBIA1300 containing GFP alone (GFP), pCAMBIA1300-SpSOS1-GFP (SpSOS1-GFP), pCAMBIA1300-SpAHA1-GFP (SpAHA1-GFP) or pCAMBIA1300-AtSOS1-GFP (AtSOS1-GFP). GFP signals from epidermal cells (left panel; scale bars = 50 μm) and mesophyll protoplasts (right panel; scale bars = 10 μm) of tobacco leaves transiently expressing either GFP alone, SpSOS1-GFP, SpAHA1-GFP or AtSOS1-GFP were recorded using confocal microscopy.

To analyze the localizations of SpSOS1 and SpAHA1 in plant cells, their ORFs fused with the *GFP* gene were each cloned into the plasmid pCAMBIA1300. AtSOS1, a plasma membrane-localized Na^+^/H^+^ antiporter [9], was used as a control. GFP-tagged SpSOS1, SpAHA1, AtSOS1 or GFP alone was transiently expressed in tobacco leaves. Confocal imaging showed that when GFP was expressed alone, a fluorescent signal was clearly observed in the cytosol and nucleus (Fig 2), which agrees with the previous observation indicating that GFP localizes to both the cytosol and nucleus [26, 27]. The GFP-fused AtSOS1 protein localized to the cell membrane (Fig 2). The fluorescence images of the SpSOS1-GFP or SpAHA1-GFP fusion protein precisely coincided with the pattern observed for the plasma membrane-localized protein AtSOS1 in tobacco leaf epidermal cells and mesophyll protoplasts, in contrast to the typical cytosolic and nuclear fluorescence distribution of GFP (Fig 2). These results indicate that both SpSOS1 and SpAHA1 are plasma membrane-bound proteins.

### Expression of SpSOS1 and SpAHA1 in *S*. *portulacastrum*

**Fig 3:**
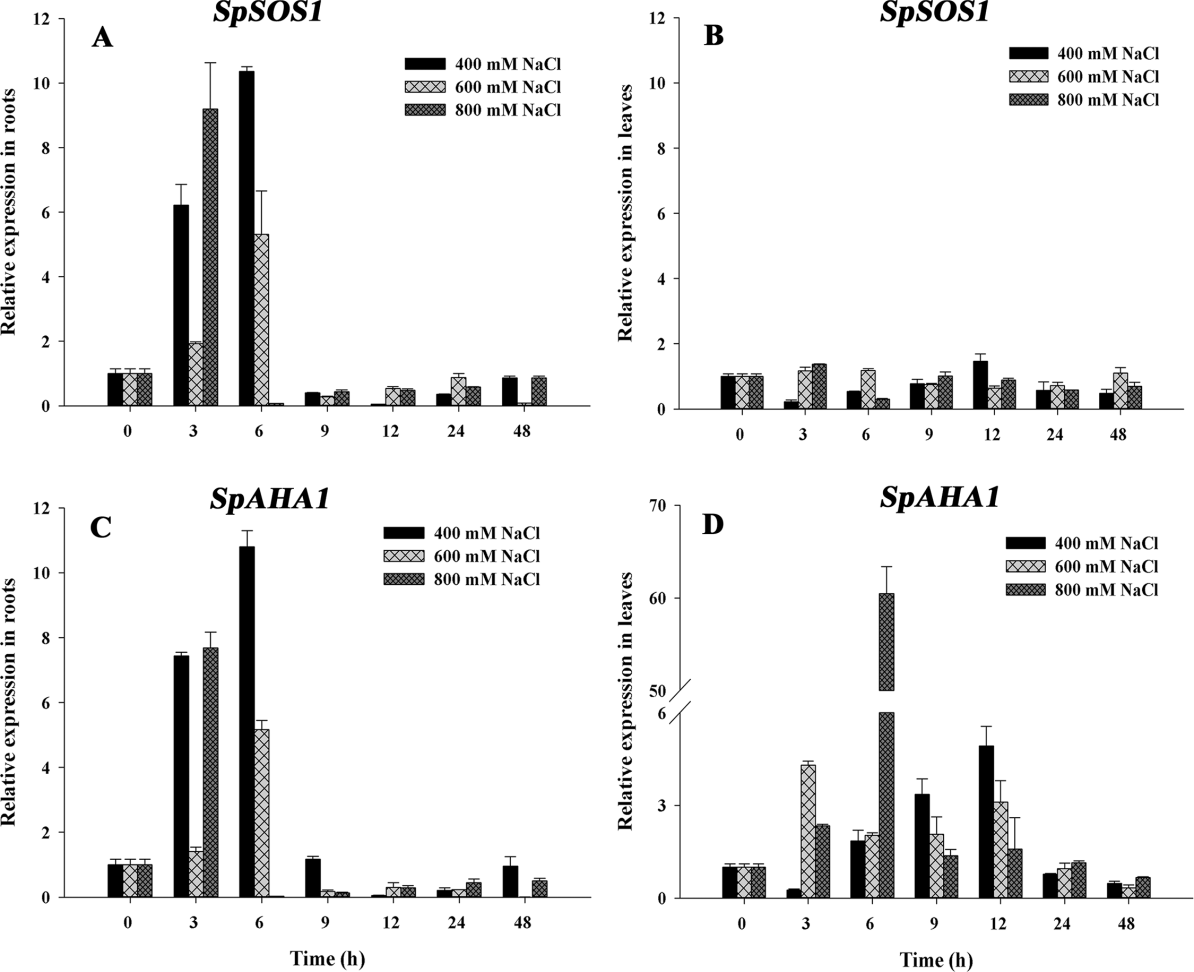
Changes in *SpSOS1* and *SpAHA1* mRNAs in *S*. *portulacastrum* exposed to salinity. Seedlings were treated with 400, 600 or 800 mM NaCl. The expression of *SpSOS1* (A, B) and *SpAHA1* (C, D) was analyzed in roots (A, C) and leaves (B, D) at different time intervals (0, 3, 6, 9, 12, 24 and 48 h) under salt treatment using real-time PCR. The expression value at the initial time (0 h) was set to 1, and the expression levels at the other time points under salinity treatment were calculated as the fold of the value at 0 h. Values are expressed as the means±SE (n = 3).

*Sesuvium portulacastrum* is a halophyte with optimal growth at 200–300 mM NaCl [14]; thus, 400 mM, 600 mM and 800 mM NaCl were chosen for treatments to assess the expression of *SpSOS1* and *SpAHA1* in *S*. *portulacastrum* seedlings under salt stress. *SpSOS1* expression in roots exhibited a consistent pattern under salt stress: all of the treatment groups exhibited a transient increase in *SpSOS1* expression in the roots that peaked at approximately 3–6 hours, after which *SpSOS1* expression gradually decreased to basal levels at 48 h (Fig 3A). In contrast, the change in *SpSOS1* expression in leaves was not significant under salinity conditions (Fig 3B). The expression pattern of *SpAHA1* in roots exposed to salt stress was similar to that of *SpSOS1* (Fig 3C). However, compared to *SpSOS1*, *SpAHA1* had a more complex leaf expression pattern under salt stress, whereby expression was initially up-regulated and then decreased (Fig 3D).

### Effect of salt stress on the growth of yeast cells

**Fig 4:**
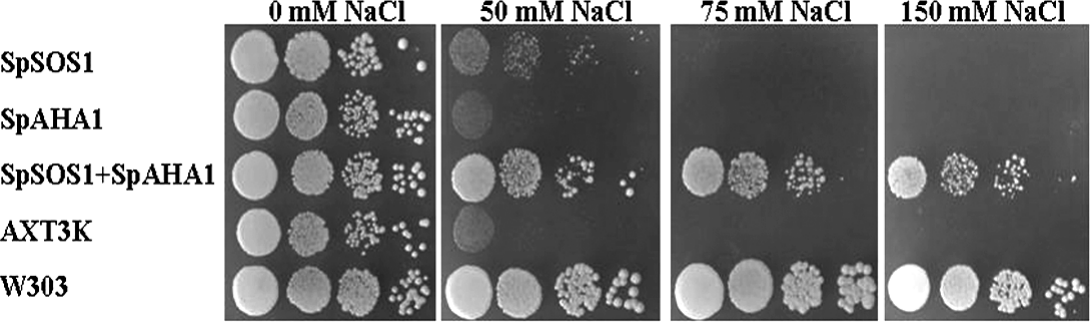
Effect of salt stress on the growth of yeast cells. The wild-type strain (W303), mutant cells (AXT3K) and recombinant strains (SpSOS1, SpAHA1 and SpSOS1+SpAHA1) were grown to saturation, and then 10-μL serial decimal dilutions were spotted onto AP plates supplemented with 0, 50, 75, or 150 mM NaCl. SpSOS1: the AXT3K strain transformed with plasmid p416-SpSOS1; SpAHA1: the AXT3K strain transformed with plasmid p414-SpAHA1; SpSOS1+SpAHA1: mutant strain AXT3K co-transformed with plasmids p416-SpSOS1 and p414-AHA1; AXT3K: untransformed mutant strain AXT3K; W303: wild-type yeast strain W303.

To investigate the roles of SpSOS1 and SpAHA1 in halotolerance, the two genes were inserted into the yeast expression vectors p416 GPD and p414 and then transformed individually or co-transformed into AXT3K. The growth of the untransformed and transgenic strains was similar under normal conditions. Untransformed yeast cells grew very poorly in the presence of NaCl, and these cells could not grow on AP medium containing 75 mM NaCl. The growth of *SpAHA1*-transgenic AXT3K was similar to that of the control strain under salt stress, whereas *SpSOS1* expression increased the salt tolerance of AXT3K. Interestingly, although the expression of *SpAHA1* alone did not influence the response of transgenic yeast cells to salt stress, AXT3K cells co-transformed with *SpSOS1* and *SpAHA1* grew better than with *SpSOS1* alone under salinity conditions. Indeed, the growth of yeast cells expressing *SpSOS1* and *SpAHA1* together was the best among the control and transgenic yeast cells and nearly reached the salt tolerance of the wild-type yeast strain W303 (Fig 4).

### Change in Na^+^ content

**Fig 5:**
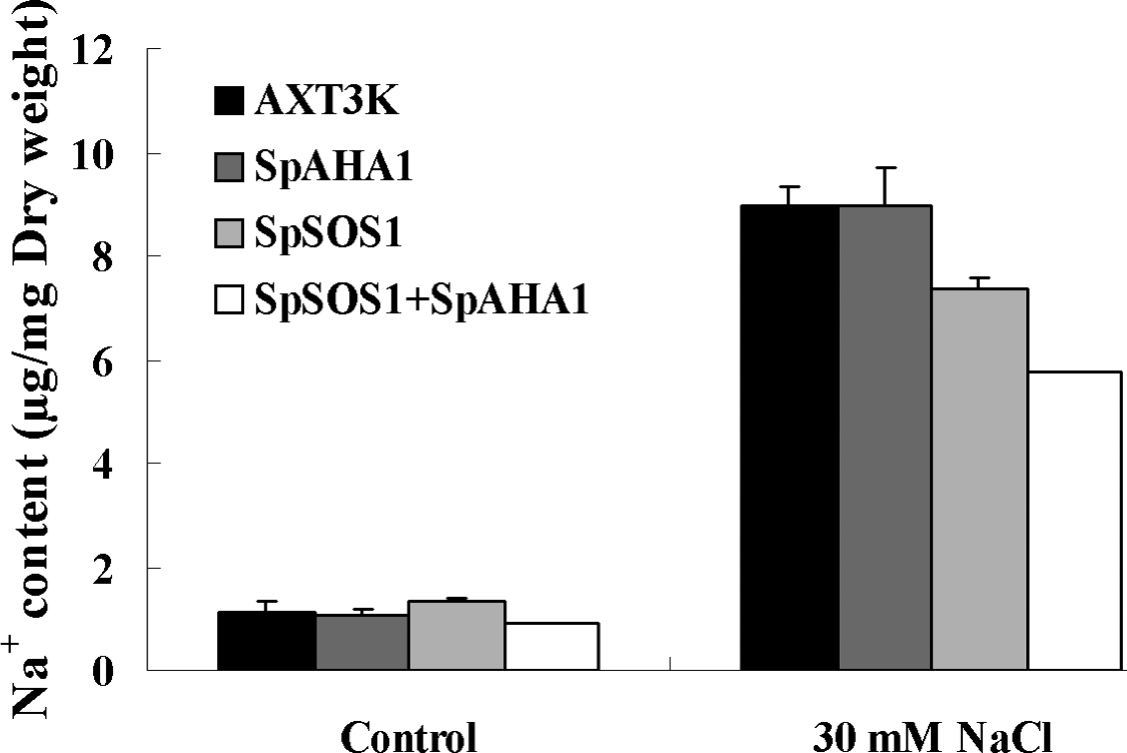
Na^+^ content in yeast cells. The Na^+^ content in unstressed yeast cells and yeast cells treated with 30 mM NaCl was measured. Values are expressed as the means±SE (n = 3). AXT3K: the non-transformed yeast strain; SpAHA1: the AXT3K strain transformed with plasmid p414-SpAHA1; SpSOS1: the AXT3K strain transformed with plasmid p416-SpSOS1; SpSOS1+SpAHA1: the mutant strain AXT3K co-transformed with plasmids p416-SpSOS1 and p414-AHA1.

The Na^+^ content in untransformed and transgenic cells was analyzed using atomic absorption spectrophotometry (Fig 5). Although the Na^+^ content did not differ between the untransformed strain and various transgenic yeast cells grown under normal conditions, NaCl treatment significantly increased the Na^+^ content in all of the yeast strains. A comparative analysis showed that the Na^+^ content increased to a higher level in the untransformed cells than in the *SpSOS1*-transgenic cells, whereas *SpAHA1* expression did not induce a significant change in the Na^+^ concentration in transgenic cells compared with untransformed cells. Interestingly, the Na^+^ content in yeast cells co-expressing *SpSOS1* and *SpAHA*1 was the lowest among all yeast strains.

### Na^+^/H^+^ exchange and H^+^-ATPase activity

**Fig 6:**
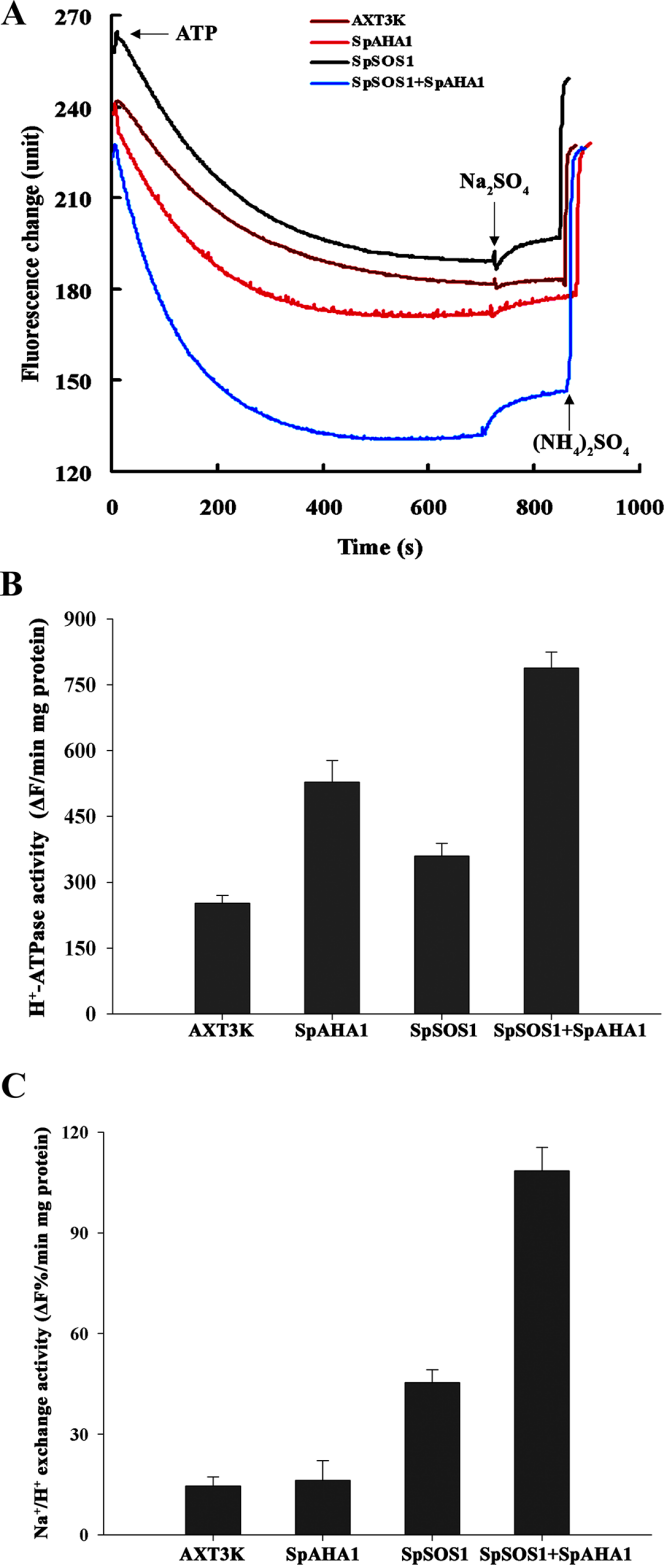
H^+^-ATPase and Na^+^/H^+^ antiport activities at plasma membrane vesicles. Plasma membranes were isolated from mutant cells (AXT3K) and related transgenic strains (SpSOS1, SpAHA1 and SpSOS1+SpAHA1) using the aqueous two-phase system. Fluorescent quenching of quinacrine was used to monitor the acidification of plasma membrane vesicles from these yeast cells. Formation of a pH gradient, indicating that H^+^ was pumped into the vesicles, was initiated with ATP. Once fluorescence was stabilized, 50 mM Na_2_SO_4_ was added to the cuvette, and fluorescence recovery, indicating Na^+^/H^+^ exchange, was monitored. The reaction was terminated by adding 25 mM (NH_4_)_2_SO_4_, which dissipated the pH gradient. The change in fluorescence is expressed as arbitrary units (A). H^+^-ATPase activity was analyzed by measuring ΔpH across the membrane; it is expressed as the change in fluorescence quenching per minute per milligrams of membrane protein (ΔF/min/mg protein) (B). Na^+^/H^+^ exchange activity is given as the proportion of dissipation of the preformed pH gradient per minute per milligram of membrane protein (ΔF%/ min/mg protein) (C). Values are expressed as the means±SE of three replicates.

The yeast strain AXT3K, which is nearly devoid of background plasma membrane Na^+^/H^+^ exchange activity, has been used to investigate the Na^+^/H^+^ exchange of some plant SOS1 proteins [19, 23, 25]. SpSOS1 suppressed the salt sensitivity of AXT3K (Fig 4). Subcellular localization showed that SpSOS1 is a plasma membrane-bound protein (Fig 2); thus, AXT3K should be a good tool for studying the Na^+^/H^+^ exchange activity of SpSOS1 at the plasma membrane. To determine whether SpSOS1 functions as a Na^+^/H^+^ exchanger in a manner similar to its counterparts AtSOS1, TaSOS1 and ThSOS1 in transgenic yeast cells [11, 19, 25], we isolated plasma membrane vesicles from AXT3K expressing the *SpSOS1* gene and from untransformed yeast cells. Little background Na^+^/H^+^ exchange activity was detected for the plasma membrane vesicles from AXT3K, which contains a mutation in the plasma membrane Na^+^/H^+^ antiporter (Fig 6), whereas the plasma membrane Na^+^/H^+^ exchange activity of yeast expressing *SpSOS1* was higher than that in untransformed yeast cells, indicating that SpSOS1 functions as a Na^+^/H^+^ antiporter. As shown in Fig 6, the generation of a transmembrane pH gradient is a prerequisite for the Na^+^-dependent movement of H^+^ out of vesicles, suggesting that if the transmembrane proton gradient increases, the Na^+^ transport capacity of SOS1 should be stronger. To test this hypothesis, the H^+^-ATPase gene *SpAHA1* was transformed into yeast cells expressing *SpSOS1*, and comparative studies were performed for Na^+^/H^+^ exchange and H^+^-ATPase activities at the plasma membrane. H^+^-pump activity was the highest for plasma membrane vesicles from the yeast cells co-expressing *SpSOS1* and *SpAHA1*, followed by that of the yeast cells individually transgenic for *SpAHA1* or *SpSOS1*; the untransformed strain displayed the lowest activity (Fig 6A and 6B). The co-transformed strain showed the highest Na^+^/H^+^ exchange activity among these tested yeast strains, and the strain transgenic for *SpSOS1* had a higher Na^+^/H^+^ exchange activity than the untransformed strain. Although plasma membrane H^+^-ATPase activity in the *SpAHA1*-transformed strain was higher than that in the untransformed cells, inconclusive results were obtained for differences in the Na^+^/H^+^ exchange activity between the *SpAHA1*-transformed strain and untransformed cells (Fig 6A and 6C).

## Discussion

One key mechanism of salinity tolerance is the ability to remove Na^+^ ions from the cytosol via plasma membrane Na^+^ transporters. SOS1 is the only plant plasma membrane Na^+^ efflux protein reported to date; thus, the protein is likely a key halotolerance factor in plants. SpSOS1 shows similarities with certain reported plasma membrane Na^+^/H^+^ antiporters, and subcellular localization analysis showed that SpSOS1 is distributed at the plasma membrane in plant cells, suggesting that SpSOS1 can regulate Na^+^ efflux and is involved in salt resistance in *S*. *portulacastrum*. SpAHA1 is a plasma membrane-bound protein and has high similarity to the *Arabidopsis* AHA family, indicating that SpAHA1 functions as a H^+^-ATPase at the plasma membrane and provides energy for metabolic processes in plants.

Real-time PCR indicated that the *SpSOS1* expression in roots increased sharply within the first 3–6 h and then decreased to basal levels under salt stress. In leaves, no obvious change in *SOS1* transcriptional abundance was found during NaCl treatment (up to 48 h). The salt-induced expression pattern of *SpSOS1* is similar to that of rice *SOS1*, which shows mRNA accumulation in roots but not in shoots under salinity [23]. In wheat, the *SOS1* expression level in leaves was not up-regulated under salinity treatment, but mRNA abundance in leaves was also substantially lower than that in roots [19]. These findings suggest that more active SOS1s can transport excess Na^+^ out of plants through the roots; correspondingly, Na^+^ transport into shoots from roots was decreased, supporting an important salt tolerance mechanism (i.e., retarding Na^+^ transport from roots to shoots). Salt stress induced the up-regulation of *SpAHA1* in both roots and leaves, indicating that the H^+^-ATPase can provide more energy for the plant to resist salinity. Interestingly, the expression patterns of *SpAHA1* and *SpSOS1* in roots are similar, suggesting that they can work in coordination. Additionally, SpSOS1 functions as a Na^+^/H^+^ antiporter using the proton gradient produced by SpAHA1.

The expression of *SpSOS1* increased the salt tolerance of transgenic AXT3K, a strain that is very sensitive to salt treatment because Na^+^ efflux transporter deficiency at the plasma membrane. Cells expressing *SpSOS1* showed higher Na^+^ efflux activity than the untransformed yeast strain, which is congruent with the lower intracellular sodium levels in the NaCl-treated yeast strain expressing *SpSOS1* compared to untransformed cells. Thus, the association between Na^+^ transport activity, intracellular Na^+^ concentration and halotolerance suggests that SpSOS1 may function as a sodium extrusion system. The plasma membrane H^+^-ATPase is very important for energizing sodium efflux, and the plasma membrane H^+^-ATPase is involved in salt tolerance in tomato [13]. In the present study, yeast cells expressing *SpAHA1* exhibited significantly increased H^+^-pump activity compared with non-transformed cells, but there were no obvious differences in either salt tolerance or Na^+^ content between *SpAHA1*-transgenic cells and non-transformed cells under salt stress. As a hypothesis, although enhanced plasma membrane H^+^ pumping in transgenic yeast cells provides extra energy to support Na^+^ export mediated by the Na^+^/H^+^ antiporter at the plasma membrane, excess Na^+^ in the cytosol cannot be transported out of cells because the Na^+^ transporter utilizing the driving force is deficient at the plasma membrane of AXT3K. This hypothesis is supported by the measurement of the Na^+^/H^+^ exchange activity of plasma membrane vesicles, showing that Na^+^/H^+^ antiport activity did not markedly increase at the plasma membrane of *SpAHA1*-transgenic cells compared with the non-transformed strain.

Although excess salt is toxic to physiological and biochemical processes in plants, some species can grow in environments containing high levels of salts because they have evolved the unique ability to resist salinity [28]. One key mechanism in salinity tolerance is the ability to remove excessive Na^+^ from the cytosol via tonoplast Na^+^/H^+^ antiporters (NHXs) and the plasma membrane Na^+^/H^+^ antiporter (SOS1). The proton motive force utilized by vacuolar Na^+^/H^+^ antiporters is generated by H^+^-translocating enzymes in the tonoplast, such as V-ATPase and H^+^-inorganic PPase. Recent studies have demonstrated that the vacuolar Na^+^/H^+^ antiporter can utilize the electrochemical H^+^ gradient generated by tonoplast H^+^-pumps to sequester Na^+^ into vacuoles via the simultaneous overexpression of the two tonoplast proteins in plants. The salt tolerance of rice co-expressing *SsNHX1* and *AtAVP1* was higher than that achieved by single-gene transformation [6], and rice plants engineered to overexpress native *NHX* and H^+^-PPase genes showed higher salt tolerance than plants expressing *OsNHX1* or *OsVP1* alone [7]. In addition, Gouiaa et al. reported that tobacco plants transformed with both *TNHXS1* and *TVP1* grew better than the single gene-transformed lines under salinity [8]. The plasma membrane Na^+^/H^+^ antiporter can also transport excess Na^+^ in the cytoplasm out of cells using the driving force of the H^+^ gradient produced by plasma membrane H^+^-pumps. *Arabidopsis* plants transformed with the *AtSOS1* gene showed higher salt tolerance than wild-type plants [12]. Although plasma membrane H^+^-ATPase activity correlates with the adaptation of plants to salt stress [13], the overexpression of plasma membrane H^+^-ATPase has not been reported to confer strong salt tolerance to transgenic plants. It is difficult to engineer organisms with vacuolar H^+^-ATPase because it comprises multiple protein subunits located in the tonoplast, and there is no experimental evidence for enhanced salt tolerance in transgenic organisms with a vacuolar H^+^-ATPase. In the present investigation, SpAHA1 produced an increased electrochemical gradient of protons to promote the Na^+^/H^+^ exchange activity of SpSOS1, with more Na^+^ ion being transported out of cells. Correspondingly, yeast cells co-expressing SpSOS1 and SpAHA1 grew better than cells transformed with SpSOS1 or SpAHA1 alone, demonstrating that the plasma membrane Na^+^/H^+^ antiporter SpSOS1 and plasma membrane H^+^-ATPase SpAHA1 could work in coordination in transgenic yeast cells. These results provide a reference for the development of more salt-tolerant crops via co-transformation of the plasma membrane Na^+^/H^+^ antiporter and H^+^-ATPase.

## Supporting Information

S1 Text The collection site of *Sesuvium portulacastrum* plants. (DOC)

## References

[1] Pardo JM, Cubero B, Leidi EO, Quintero FJ. Alkali cation exchangers: roles in cellular homeostasis and stress tolerance. J Exp Bot. 2006; 57(5): 1181–1199. 16513813 10.1093/jxb/erj114

[2] Apse MP, Aharon GS, Snedden WA, Blumwald E. Salt tolerance conferred by overexpression of a vacuolar Na^+^/H^+^ antiport in *Arabidopsis* . Science. 1999; 285(5431):1256–1258. 10455050 10.1126/science.285.5431.1256

[3] Gaxiola RA, Rao R, Sherman A, Grisafi P, Alper SL, Fink GR. The *Arabidopsis thaliana* proton transporters, AtNhx1 and Avp1, can function in cation detoxification in yeast. Proc Natl Acad Sci USA. 1999; 96(4): 1480–1485. 9990049 10.1073/pnas.96.4.1480 PMC15488

[4] Rodriguez-Rosales MP, Galvez FJ, Huertas R, Aranda MN, Baghour M, Cagnac O, et al Plant NHX cation/proton antiporters. Plant Signal Behav. 2009; 4(4): 265–276. 19794841 10.4161/psb.4.4.7919 PMC2664485

[5] Gaxiola RA, Li J, Undurraga S, Dang LM, Allen GJ, Alper SL, et al Drought- and salt-tolerant plants result from overexpression of the AVP1 H^+^-pump. Proc Natl Acad Sci USA. 2001; 98: 11444–11449. 11572991 10.1073/pnas.191389398 PMC58749

[6] Zhao FY, Zhang XJ, Li PH, Zhao YX, Zhang H. Co-expression of the *Suaeda salsa SsNHX1* and *Arabidopsis AVP1* confer greater salt tolerance to transgenic rice than the single SsNHX1. Mol Breeding. 2006; 17(4): 341–353.

[7] Liu SP, Zheng LQ, Xue YH, Zhang Q, Wang L, Shou HX. Overexpression of *OsVP1* and *OsNHX1* increases tolerance to drought and salinity in rice. J Plant Biol. 2010; 53: 444–452.

[8] Gouiaa S, Khoudi H, Leidi EO, Pardo JM, Masmoudi K. Expression of wheat Na^+^/H^+^ antiporter *TNHXS1* and H^+^-pyrophosphatase *TVP1* genes in tobacco from a bicistronic transcriptional unit improves salt tolerance. Plant Mol Biol. 2012; 79: 137–155. 10.1007/s11103-012-9901-6 22415161

[9] Qiu QS, Guo Y, Dietrich MA, Schumaker KS, Zhu JK. Regulation of SOS1, a plasma membrane Na^+^/H^+^ exchanger in *Arabidopsis thaliana*, by SOS2 and SOS3. Proc Nat Acad Sci USA. 2002; 99: 8436–8441. 12034882 10.1073/pnas.122224699 PMC123085

[10] Quintero FJ, Ohta M, Shi H, Zhu JK, Pardo JM. Reconstitution in yeast of the *Arabidopsis* SOS signalling pathway for Na^+^ homeostasis. Proc Nat Acad Sci USA. 2002; 99: 9061–9066. 12070350 10.1073/pnas.132092099 PMC124423

[11] Quintero FJ, Martı´nez-Atienza J, Caros I, Jiang XY, Kim WY, Ali Z, et al Activation of the plasma membrane Na/H antiporter SOS1 by phosphorylation of an auto-inhibitory C-terminal domain. Proc Nat Acad Sci USA. 2011; 108: 2611–2616. 10.1073/pnas.1018921108 21262798 PMC3038701

[12] Yang Q, Chen ZZ, Zhou XF, Yin HB, Li X, Xin XF, et al Overexpression of *SOS* (Salt Overly Sensitive) genes increases salt tolerance in transgenic *Arabidopsis* . Mol Plant. 2009; 2: 22–31. 10.1093/mp/ssn058 19529826 PMC2639737

[13] Kerkeb L, Donaire JP, Venema K, Rodriguez-Rosales MP. Tolerance to NaCl induces changes in plasma membrane lipid composition, fluidity and H^+^-ATPase activity of tomato calli. Physiol Plant. 2001; 113: 217–224. 12060299 10.1034/j.1399-3054.2001.1130209.x

[14] Yi XP, Sun Y, Yang Q, Guo AP, Chang LL, Wang D, et al Quantitative proteomics of *Sesuvium portulacastrum* leaves revealed that ion transportation by V-ATPase and sugar accumulation in chloroplast played crucial roles in halophyte salt tolerance. J Proteomics. 2014; 99: 84–100. 10.1016/j.jprot.2014.01.017 24487036

[15] Yang CL, Duan RJ, Li RM, Hu XW, Fu SP, Guo JC. The physiological characteristics of salt-tolerance in *Sesuvium portulacastrum* L. Acta Ecologica Sinica. 2010; 30(17): 4617–4627.

[16] Waadt R, Schmidt LK, Lohse M, Hashimoto K, Bock R, Kudla J. Multicolor bimolecular fluorescence complementation reveals simultaneous formation of alternative CBL/CIPK complexes *in planta* . Plant J. 2008; 56: 505–516. 10.1111/j.1365-313X.2008.03612.x 18643980

[17] Yoo SD, Cho YH, Sheen J. *Arabidopsis* mesophyll protoplasts: a versatile cell system for transient gene expression analysis. Nat Protocols. 2007; 2(7): 1565–1572. 17585298 10.1038/nprot.2007.199

[18] Xu YY, Zhou Y, Hong S, Xia ZH, Cui DQ, Guo JC, et al Functional characterization of a wheat NHX antiporter gene *TaNHX2* that encodes a K^+^/H^+^ exchanger. Plos One. 2013; 8(11): e78098 10.1371/journal.pone.0078098 24223765 PMC3815223

[19] Xu HX, Jiang XY, Zhan KH, Cheng XY, Chen XJ, Pardo JM, et al Functional characterization of a wheat plasma membrane Na^+^/H^+^ antiporter in yeast. Arch Biochem Biophys. 2008; 473: 8–15. 10.1016/j.abb.2008.02.018 18316035

[20] Menendez A, Larsson C, Ugalde U. Purification of functionally sealed cytoplasmic side-out plasma membrane vesicles from *Saccharomyces cerevisias* . Anal Biochem. 1995; 230(2): 308–314. 7503423 10.1006/abio.1995.1479

[21] Hong S, Cong XL, Jing HY, Xia ZH, Huang X, Hu XW, et al Characterization of Ca^2+^/H^+^ exchange in the plasma membrane of Saccharomyces cerevisiae. Arch Biochem Biophys. 2013; 537: 125–132. 10.1016/j.abb.2013.07.005 23871844

[22] Brandford MM. A rapid and sensitive method for the quantitation of microgram quantities of protein utilizing the principle of protein-dye binding. Anal Biochem. 1976; 72: 248–254. 942051 10.1016/0003-2697(76)90527-3

[23] Martinez-Atienza J, Jiang XY, Garciadeblas B, Mendoza I, Zhu JK, Pardo JM, et al Conservation of the salt overly sensitive pathway in rice. Plant Physiol. 2007; 143: 1001–1012. 17142477 10.1104/pp.106.092635 PMC1803719

[24] Tang RJ, Liu H, Bao Y, Lv QD, Yang L, Zhang HX. The woody plant poplar has a functionally conserved salt overly sensitive pathway in response to salinity stress. Plant Mol boil. 2010; 74: 367–380. 10.1007/s11103-010-9680-x 20803312

[25] Oh DH, Leidi E, Zhang Q, Hwang SM, Li YZ, Quintero FJ, et al Loss of halophytism by interference with SOS1 expression. Plant Physiol. 2009; 151: 210–222. 10.1104/pp.109.137802 19571313 PMC2735974

[26] Kim BG, Waadt R, Cheong YH, Pandey GK, Dominguez-Solis JR, Schultke S, et al The calcium sensor CBL10 mediates salt tolerance by regulating ion homeostasis in *Arabidopsis* . Plant J. 2007; 52: 473–484. 17825054 10.1111/j.1365-313X.2007.03249.x

[27] D’Angelo C, Weinl S, Batistic O, Pandey GK, Cheong YH, Schultke S, et al Alternative complex formation of the Ca-regulated protein kinase CIPK1 controls abscisic acid-dependent and independent stress responses in *Arabidopsis* . Plant J. 2006; 48: 857–872. 17092313 10.1111/j.1365-313X.2006.02921.x

[28] Flowers TJ, Colmer TD. Salinity tolerance in halophytes. New Phytol. 2008; 179: 945–963. 10.1111/j.1469-8137.2008.02531.x 18565144

