# Supplementary material for: SpAHA1 and SpSOS1 Coordinate in Transgenic Yeast to Improve Salt Tolerance
Source: PLoS One. 2015 Sep 4;10(9):e0137447. doi: 10.1371/journal.pone.0137447 (PMC4560418; doi:10.1371/journal.pone.0137447)
Supplement: S1 Text — (DOC) [file pone.0137447.s001.doc]

**S1 Text. The collection site of *Sesuvium portulacastrum* plants**

*Sesuvium portulacastrum* plants grow naturally in the seashore of Haikou, China. The collection site of *S. portulacastrum* plants in the seashore is a wasteland, so no one is responsible for this field. Furthermore, *Sesuvium portulacastrum* grows worldwide and is not specifically exploited or an endangered or protected species. Therefore, no specific permission was required for the collection of the plants in the field.
